# Umgang mit assistiertem Suizid – Kenntnisse, Einstellungen und Erfahrungen von Mitgliedern der Deutschen Gesellschaft für Palliativmedizin

**DOI:** 10.1007/s00103-024-03960-z

**Published:** 2024-10-07

**Authors:** Jacqueline Schwartz, Yann-Nicolas Batzler, Manuela Schallenburger, Alexandra Scherg, Jonas Jansen, Stefan Meier, Remo Küppers, Heiner Melching, Ulrich Grabenhorst, Wiebke Nehls, Claudia Bausewein, Martin Neukirchen

**Affiliations:** 1 Universitätsklinikum Düsseldorf, Interdisziplinäres Zentrum für Palliativmedizin, Heinrich-Heine-Universität Düsseldorf, Moorenstr. 5, 40225 Düsseldorf, Deutschland; 2 Centrum für Integrierte Onkologie Aachen Bonn Cologne Düsseldorf (CIO ABCD), Aachen, Bonn, Köln, Düsseldorf, Deutschland; 3 Palliativmedizin, 2. Medizinische Klinik, Universitäres Cancer Center Hamburg, Universitätsklinikum Hamburg-Eppendorf, Hamburg, Deutschland; 4 Klinik für Akut- und Notfallmedizin, Rheinland Klinikum, Lukaskrankenhaus, Neuss, Deutschland; 5 Klinik für Anästhesiologie, Universitätsklinikum Düsseldorf, Heinrich-Heine-Universität Düsseldorf, Düsseldorf, Deutschland; 6 Deutsche Gesellschaft für Palliativmedizin, Berlin, Deutschland; 7 SAPV-Team HomeCare Linker Niederrhein gGmbH, Viersen, Deutschland; 8 Klinik für Palliativmedizin und Geriatrie, Helios Klinikum Emil von Behring, Berlin, Deutschland; 9 Klinik und Poliklinik für Palliativmedizin, LMU Klinikum München, München, Deutschland

**Keywords:** Deutsche Gesellschaft für Palliativmedizin, Assistierter Suizid, Todeswunsch, § 217 StGB, Einstellungen von Gesundheitspersonal, German Association for Palliative Medicine, Assisted suicide, Desire to die, § 217 German Criminal Code, Attitude of health personnel

## Abstract

**Hintergrund:**

Im Februar 2020 erklärte das Bundesverfassungsgericht § 217 StGB für nichtig. Seitdem ist die geschäftsmäßige Förderung der Selbsttötung straffrei. Ziel dieser Studie ist die Beschreibung von Kenntnissen, Einstellungen und Erfahrungen von Mitgliedern der Deutschen Gesellschaft für Palliativmedizin (DGP) im Umgang mit Todes- und Suizidwünschen.

**Methoden:**

Online-Befragung aller DGP-Mitglieder von 07–09/2023 mittels Qualtrics®. Der Fragebogen wurde anhand aktueller Literatur entwickelt und nach einer ersten Anwendung unter jungen Mediziner*innen und einer interprofessionellen Expertenrunde mit Konsensabstimmung angepasst. Die Auswertung erfolgte deskriptiv und explorativ.

**Ergebnisse:**

991 DGP-Mitglieder (18 %) nahmen teil, davon 57,0 % Ärzt*innen (*n* = 545/957) und 23,4 % Pflegende (*n* = 224/957). 197/851 (23,1 %) gaben falsch an, der assistierte Suizid sei in Deutschland standesrechtlich verboten. 430/914 (47,1 %) gaben an, dass sich Palliativteams nicht nur auf Maßnahmen zur Suizidprävention beschränken sollen. In palliativen Behandlungssituationen konnten sich 473/926 (51,5 %) die Mitwirkung an Suizidassistenz vorstellen. Für Situationen, in denen der Gesundheitszustand nicht ausschlaggebend ist, lehnten 766/930 (82,4 %) eine Mitwirkung ab. 71 % wünschten sich eine gesetzliche Regelung der Suizidassistenz.

**Diskussion:**

Bei den Teilnehmenden bestehen Wissenslücken bzgl. straf- und standesrechtlicher Einordnung des assistierten Suizids. Weitere Aufklärungsarbeit ist notwendig. Die Teilnehmenden können sich eine Suizidassistenz eher bei palliativ Erkrankten vorstellen. Wie in Befragungen unter Mitgliedern anderer Fachgesellschaften bilden sich Einstellungen erfahrener Mitarbeiter*innen ab. Sie haben im Vergleich zu jüngeren Ärzt*innen eine eher restriktive Haltung im Hinblick auf die Suizidassistenz.

## Abstract (en)

**Background:**

In February 2020, the Federal Constitutional Court declared § 217 of the German Criminal Code void. Ever since, assisted suicide services have been legal in Germany. This study aims to describe the knowledge, attitudes and experiences of members of the German Association for Palliative Medicine (DGP) regarding assisted suicide.

**Methods:**

Online survey with members of the DGP from July to September 2023 using Qualtrics®. The study group developed the questionnaire based on current literature; it was adapted following an initial application among young physicians and an interprofessional panel of experts with consensus voting. Data was analysed using descriptive and explorative statistics.

**Results:**

991 DGP-members (18%) participated, of which physicians made up 57.0% (*n* = 545/957) and nurses 23.4% (*n* = 224/957). Of the participants, 197/851 (23.1%) incorrectly stated that assisted suicide is prohibited by professional code, 430/914 (47.1%) rejected a restriction of palliative care teams to suicide prevention measures, and 766/930 (82.4%) rejected personal involvement in assisted suicide regardless of a patient’s health status. For patients in palliative situations, 473/926 (51.1%) could imagine participating in assisted suicide, and 71% wanted new legislation regulating assisted suicide.

**Conclusion:**

There are gaps in the knowledge of the participating members of the DGP regarding the legal and professional status of assisted suicide. Further educational work is needed in this regard. The participants can more easily imagine assisted suicide for people in palliative disease trajectories. As in surveys of members of other medical societies, the attitudes of more experienced staff are reflected. Compared to younger healthcare professionals, they have a more restrictive attitude towards the concept of assisted suicide.

## Einleitung

Suizidassistenz war in Deutschland in der Vergangenheit legal, eine geschäftsmäßige, das heißt eine auf Wiederholung angelegte Förderung, z. B. durch Organisationen, Vereine und Einzelpersonen, war durch den § 217 StGB seit 2015 jedoch strafbewehrt. Im Jahr 2020 erklärte das Bundesverfassungsgericht diesen Paragrafen für nichtig und öffnete die Suizidassistenz für alle Bürger*innen mit Verweis auf das Persönlichkeitsrecht [1]. In Deutschland herrscht somit bezogen auf Möglichkeiten der Suizidassistenz eine der liberalsten Situationen weltweit. In der Folge des Urteils des Bundesverfassungsgerichts wollten 2 fraktionsübergreifende Gruppen die Ausgestaltung des Urteils durch eine gesetzliche Regelung erreichen. Jedoch scheiterten beide Gesetzesentwürfe im Juli 2023 im Bundestag. Parallel wurde ein Antrag auf gesetzliche Verankerung der Suizidprävention verabschiedet. Als Konsequenz des Urteils des Bundesverfassungsgerichtes änderte die Bundesärztekammer 2021 auf dem 124. Deutschen Ärztetag die Musterberufsordnung und strich das Verbot der Suizidassistenz. Eine Beschlussempfehlung des 127. Deutschen Ärztetages sieht nun folgende Formulierung vor: „Die Mitwirkung bei der Selbsttötung (assistierter Suizid) ist grundsätzlich keine ärztliche Aufgabe. Sie ist bei schwerer oder unerträglicher Erkrankung nach wohlabgewogener Gewissensentscheidung im Einzelfall zulässig“ [2, 3].

Mit Urteil des Bundesverfassungsgerichtes wurde eine breite gesellschaftliche Debatte angestoßen, die weiter andauert und insbesondere jene medizinischen Fachkreise beschäftigt, die mit der Behandlung von lebenslimitiert erkrankten Menschen betraut sind. In diese Debatte fließen u. a. moralisch-ethische Perspektiven, straf- wie standesrechtliche Fragestellungen sowie Rollenverständnisse unterschiedlicher Berufsgruppen ein. Im Rahmen der Betreuung schwerkranker und sterbender Patient*innen sind Mitarbeiter*innen multiprofessionell zusammengesetzter Palliativteams im Alltag wiederkehrend mit Todes- und Suizidwünschen konfrontiert [4]. In der erweiterten S3-Leitlinie Palliativmedizin für Patient*innen mit einer nicht heilbaren Krebserkrankung aus dem Jahr 2020 widmet sich daher ein ganzes Kapitel dem Thema Todeswünsche [5]. Neben der Bundesärztekammer [2] oder dem Deutschen Hospiz- und Palliativverband [6] hat sich auch die Deutsche Gesellschaft für Palliativmedizin (DGP) intensiv mit dem Umgang mit Todes- und Suizidwünschen auseinandergesetzt [7].

Anhaltend wird die Debatte um Suizidassistenz und angrenzende Bereiche wie den freiwilligen Verzicht auf Essen und Trinken (FVET) oder die Tötung auf Verlangen von kontroversen Diskussionen und individuell sehr unterschiedlichen Meinungen unter palliativmedizinisch tätigen Mitarbeiter*innen geprägt. Nach unserem Kenntnisstand liegt bisher kein Stimmungsbild der mehr als 6000 Mitglieder der DGP vor. Ziel der vorliegenden Studie war es, Kenntnisse, Einstellungen und Erfahrungen von Mitgliedern der DGP im Umgang mit Todes- und Suizidwünschen abzubilden.

## Methoden

Die Studienbeschreibung folgt der CHERRIES-Checkliste für Online-Umfragen.

### Design.

Es wurde eine Online-Umfrage unter Mitgliedern der DGP mittels des Online-Umfragetools Qualtrics® durchgeführt.

### Entwicklung des Fragebogens und Pretest.

Bei der Befragung waren neben demografischen Daten insbesondere folgende Themen von Interesse: Kenntnisstand hinsichtlich der gesetzlichen Regelung, Umgang mit Todeswunschäußerungen, Ansprechpartner*innen für Suizidwünsche, Erfahrungen mit Suizidassistenz sowie Stellenwert suizidpräventiver Maßnahmen bei Palliativpatient*innen. Der Fragebogen, der ausgewählte Aspekte der Debatte zur Suizidassistenz abbildet, wurde in der Studiengruppe auf Basis einer Literaturrecherche entwickelt. Die Fragen wurden in einer interdisziplinären und multiprofessionellen Expertengruppe abgestimmt und durch Prätests auf Inhalt und Verständlichkeit geprüft. Daraufhin fand der Fragebogen eine erste Anwendung im Rahmen einer Befragung unter jungen Intensiv- und Notfallmediziner*innen [8]. Im Folgenden wurden nach einer interprofessionellen Expertenrunde mit Konsensabstimmung diskussionswürdige Fragen angepasst (Fragen zur möglichen Zuständigkeit verschiedener Berufsgruppen), erweitert (Fragen zur Jurisdiktion) oder gestrichen, wenngleich großer Wert darauf gelegt wurde, die gestellten Fragen möglichst nicht zu verändern, um die Vergleichbarkeit nicht zu gefährden. Um Verzerrungen durch ausschließliche Selbsteinschätzung der Kenntnisse auszuschließen, wurden konkrete Wissensfragen ergänzt. Demografische Daten wurden über Multiple-Choice- und Multiple-Select-Fragen erhoben. Die Fragen konnten anhand einer 5‑Punkt-Likert-Skala beantwortet werden, wobei die Aussagen „trifft voll zu“ und „trifft eher zu“ als Zustimmung und die Aussagen „trifft eher nicht zu“ und „trifft nicht zu“ als Ablehnung gewertet wurden. Eine quantitative Einschätzung wurde mit der Unterteilung „noch nie“, „vereinzelt (2 oder weniger Patient*innen/Fälle)“, „gelegentlich (2 bis 10 Patient*innen/Fälle)“, „häufig (10–50 Patient*innen/Fälle)“ und „sehr häufig (mehr als 50 Patient*innen/Fälle)“ möglich. Des Weiteren erfolgte die Abfrage nach Mitwirkung z. B. bei Suizidassistenz anhand dichotomer Fragen.

### Rekrutierungsprozess, Studienpopulation und Studienadministration.

Um möglichst viele Mitglieder der DGP zu erreichen, wurde der Teilnahmelink am 10.07.2023 per Mitlieder-Newsletter an 5500 digital erreichbare Mitglieder der insgesamt 6386 Mitglieder (Stand 15.09.2023) versandt. Es wurde außerdem am 10.08.2023 und am 31.08.2023 eine Reminder-E-Mail an die gleiche Gruppe verschickt. Die Bearbeitung des Fragebogens war bis zum September 2023 über die Plattform Qualtrics möglich. Die Bearbeitung war freiwillig. Pro Seite wurden maximal 10 Fragen gestellt, der Fragebogen erstreckte sich insgesamt über 7 Seiten. Mithilfe der CHERRIES-Kriterien wurde ermittelt, in welchem Maße der Fragebogen angesehen sowie teilweise oder komplett bearbeitet wurde [9].

### Datenanalyse.

Nach systematischer Aufbereitung wurden die Daten mit Microsoft Excel für Mac, Version 16.81 (Microsoft Inc., Redmond, WA, USA), und SPSS Statistics 29.02 (IBM Inc., Armonk, NY, USA) deskriptiv analysiert. Zur Beurteilung der Repräsentativität der Teilnehmenden in Bezug auf Alter, Geschlecht und Profession im Vergleich zur Grundgesamtheit der Mitglieder der DGP wurde ein Mann-Whitney-U-Test durchgeführt. Es folgten explorative Analysen einiger Items zur Überprüfung eines professionsabhängigen Einflusses auf die Haltungen zur Suizidassistenz. Korrelationsmaße wurden mittels Spearman (ordinal vs. ordinal) oder Cramers V (nominal vs. ordinal) ermittelt, mögliche Zusammenhänge bzw. Unterschiede mittels Χ^2^-Test. Relevante Freitextantworten wurden in Kategorien zusammengefasst und deskriptiv analysiert.

## Ergebnisse

Bis zum Abschluss der Befragung im September 2023 öffneten 2530 Mitglieder die Newsletter, 1296 Teilnehmende öffneten die Umfrage (View Rate: 51,2 %). Von dieser Gruppe nahmen 991 Befragte teil (Participation Rate: 76,5 %), 921 von ihnen bearbeiteten den Fragebogen bis zur letzten Seite (Completion Rate: 92,9 %), 660 von ihnen vollständig (Completeness Rate 66,6 %). Unvollständig beantwortete Fragebögen wurden ebenfalls in die Auswertung aufgenommen. Insgesamt nahmen somit 18 % der per E‑Mail erreichbaren DGP-Mitglieder teil.

### Soziodemografische Daten.

In der Befragung ordneten sich 558/908 (61,5 %) der Teilnehmenden dem weiblichen Geschlecht und 319/908 (35,1 %) dem männlichen Geschlecht zu. Von den Befragten waren mit 545/957 Teilnehmenden Ärzt*innen (57,0 %) die größte Berufsgruppe, gefolgt von Pflegenden (*n* = 224/957, 23,4 %) und weiteren Professionen, wie z. B. Psycho(onko)log*innen (*n* = 47/957, 4,9 %), Sozialarbeiter*innen (*n* = 31/957, 3,2 %) und Seelsorgende (*n* = 16/957, 1,7 %). Dies entspricht in etwa dem Verhältnis der Professionen innerhalb der Fachgesellschaft (Ärzt*innen 56,5 %, Pflegende 27,9 %). 333/909 (36,6 %) der Mitglieder gehörten der größten Altersgruppe der 51- bis 60-Jährigen an. 645/893 (72,2 %) der Befragten gaben an, mehr als 18 Jahre Berufserfahrung zu haben. 22/893 (2,5 %) der Befragten hatten weniger als 4 Jahre Berufserfahrung. 513/896 (57,3 %) der Teilnehmenden waren mehr als 8 Jahre in der Palliativversorgung tätig. Weitere demografische Daten sind Tab. 1 zu entnehmen. Im Hinblick auf Geschlecht und Profession waren die Teilnehmenden repräsentativ für die Gesamtheit aller Mitglieder der DGP. Lediglich für die Altersverteilung ergab sich ein signifikanter Unterschied (*p* = 0,04).

**Tab. 1:** Soziodemografische Daten der Teilnehmenden im Vergleich zu den Mitgliedern der Deutschen Gesellschaft für Palliativmedizin (DGP) insgesamt

	Teilnehmende *n* [%]	Mitglieder DGP 2024 *n* [%] *n* = 6360
**Geschlecht**^a^		
Weiblich	558/908 [61,5]	4068 [64,0]
Männlich	319/908 [35,1]	2269 [35,7]
Divers	2/908 [0,2]	0^d^
Keine Angabe^b^	29/908 [3,2]	23 [0,4]
**Alter**		
20–30 Jahre	25/909 [2,8]	93 [1,5]
31–40 Jahre	89/909 [9,8]	511 [8,0]
41–50 Jahre	218/909 [23,9]	1342 [21,1]
51–60 Jahre	333/909 [36,6]	2184 [34,3]
> 60 Jahre	227/909 [25,0]	2230 [35,1]
Keine Angabe^b^	17/909 [1,9]	–
**Berufsgruppe**		
Ärzt*innen	545/957 [57,0]	3592 [56,5]
Pflegende	224/957 [23,4]	1773 [27,9]
Physiotherapeut*innen	6/957 [0,6]	110 [1,7]
Psycho(onko)log*innen	47/957 [4,9]	132 [2,1]
Mitarbeitende im Bereich soziale Arbeit	31/957 [3,2]	188 [3,0]
Seelsorgende	16/957 [1,7]	111 [1,7]
Andere	88/957 [9,2]	454 [7,1]
**Zusatzbezeichnungen/Fort- und Weiterbildungen**^**c**^		
Palliativmedizin (Medizin)	511/1465 [34,9]	–
Palliative Care (Pflege)	322/1465 [22,0]	–
Pflege in der Onkologie	24/1465 [1,6]	–
Anästhesie und Intensivpflege	29/1465 [2,0]	–
Notfallmedizin	166/1465 [11,3]	–
Intensivmedizin	82/1465 [5,6]	–
Onkologie	72/1465 [4,9]	–
Geriatrie	61/1465 [4,2]	–
Andere	198/1465 [13,5]	–
**Berufserfahrung gesamt**		
< 4 Jahre	22/893 [2,5]	–
4–8 Jahre	30/893 [3,4]	–
9–13 Jahre	87/893 [9,7]	–
14–18 Jahre	109/893 [12,2]	–
> 18 Jahre	645/893 [72,2]	–
**Tätigkeit in der Palliativversorgung**		
< 4 Jahre	106/896 [11,8]	–
4–8 Jahre	160/896 [17,9]	–
9–13 Jahre	181/896 [20,2]	–
14–18 Jahre	155/896 [17,3]	–
> 18 Jahre	177/896 [19,8]	–
Aktuell nicht in der Palliativversorgung tätig	117/896 [13,1]	–

^a^ Geschlechteranteile in der Bevölkerung der BRD (2022) zum Vergleich: weiblich: 50,7 %, männlich: 49,2 %, divers: < 0,1 % (Quelle: https://www.zensus2022.de/DE/Aktuelles/Demografie_VOE.html)

^b^ Mögliche Antwortoption

^c^ Mehrfachantwort möglich

^d^ Diskrepanz ggf. durch nicht erfolgte Anpassung persönlicher Daten bei DGP-Mitgliedschaft bedingt

### Wissensstand und Kenntnisse.

Kenntnisse im Hinblick auf die Unterscheidung von assistiertem Suizid, freiwilligem Verzicht auf Essen und Trinken (FVET) sowie Tötung auf Verlangen wurden durch Selbsteinschätzung erhoben. 927/990 (98, 0 %) der Befragten gaben an, alle 3 Modalitäten voneinander abgrenzen zu können. Weitere Ergebnisse zur Selbsteinschätzung der Kenntnisse sind in Tab. 2 dargestellt. Mithilfe von Fragen, die das Wissen zur Jurisdiktion explizit erfragen, ergab sich, dass 60/851 (7,1 %) der Befragten den assistierten Suizid fälschlich als Straftat einordneten und 197/851 (23,1 %) davon ausgingen, er sei standesrechtlich verboten. In den Subgruppenanalysen für Ärzt*innen, Pflegende und die anderen Professionen ergaben sich bzgl. des Wissens keine signifikanten Unterschiede. Nach Ablehnung zweier Gesetzesvorschläge im Bundestag zur Regulation des assistierten Suizids im Sommer 2023 wünschten sich 696/974 (71,0 %) der Befragten eine neue gesetzliche Regelung. Als häufigster Grund wurde von 185/660 (43,0 %) der Befragten der Wunsch nach mehr Rechtssicherheit angegeben. 25,0 % gaben an, dass sie sich durch eine gesetzliche Regelung einen Schutz der Suizidwilligen wünschen, sollte keine Freiverantwortlichkeit zugrunde liegen. 24,0 % wünschten sich eine unabhängige Überprüfung der Freiverantwortlichkeit.

**Tab. 2:** Kenntnisse der Teilnehmenden zur Unterscheidung von assistiertem Suizid, freiwilligem Verzicht auf Essen und Trinken (FVET) sowie Tötung auf Verlangen (in Deutschland verboten)

	Trifft voll zu *n* [%]	Trifft eher zu *n* [%]	Weder noch *n* [%]	Trifft eher nicht zu *n* [%]	Trifft gar nicht zu *n* [%]
Q3: Ich kann definieren, was *freiwilliger Verzicht auf Essen und Trinken (FVET)* ist (*n* = 991)	793 [80,0]	182 [18,4]	6 [0,6]	7 [0,7]	3 [0,3]
Q4: Ich kann definieren, was *assistierter Suizid* ist (*n* = 991)	736 [74,3]	233 [23,5]	6 [0,6]	13 [1,3]	3 [0,3]
Q5: Ich kann definieren, was *Tötung auf Verlangen* ist (*n* = 991)	786 [79,3]	185 [18,7]	8 [0,8]	10 [1,0]	2 [0,2]
Q6: Mir sind die *Unterschiede der Begriffe *freiwilliger Verzicht auf Essen und Trinken (FVET)/assistierter Suizid/Tötung auf Verlangen klar (*n* = 990)	773 [78,1]	199 [20,1]	2 [0,2]	13 [1,3]	3 [0,3]

### Einstellungen.

473/926 (51,0 %) der Teilnehmenden gaben an, sich eine persönliche Mitwirkung beim assistierten Suizid nur bei Patient*innen in palliativen Behandlungssituationen vorstellen zu können. Die Frage, ob sich die Teilnehmenden eine persönliche Mitwirkung beim assistierten Suizid unabhängig vom Gesundheitszustand der Menschen mit Suizidwunsch vorstellen können, beantworteten 766/930 (82,0 %) der Befragten ablehnend, 129/930 (13,9 %) beantworteten diese Frage akzeptierend.

Von den Befragten lehnten 839/928 (90,0 %) eine in Deutschland rechtlich verbotene Mitwirkung bei einer Tötung auf Verlangen ab und 66/928 (7,0 %) konnten sich eine persönliche Mitwirkung vorstellen.

### Ansprechpartner*innen für Todes- und Suizidwünsche.

Von den Befragten gaben 354/926 (38,0 %) an, sehr häufig (> 50 Patient*innen), 304/926 (33,0 %) häufig (11 bis 50 Patient*innen) und 169/926 (18,2 %) gelegentlich (3 bis 10 Patient*innen) Patient*innen mit Todeswunschäußerungen betreut zu haben. Auf die Frage, wie häufig die Teilnehmenden ihre Patient*innen aktiv auf das Vorhandensein von Todeswünschen angesprochen haben, antworteten 262/926 (28,0 %) mit „sehr häufig“ (> 50 Patient*innen), 228/926 (25,0 %) mit „häufig“ (11–50 Patient*innen), 205/926 (22,0 %) mit „gelegentlich“ (3–10 Patient*innen), 140/926 (15,0 %) mit „vereinzelt“ (2 oder weniger Patient*innen) und 91/926 (10,0 %) mit „nie“.

Im Vergleich zwischen Ärzt*innen und Pflegenden haben 37,7 % der Ärzt*innen Patient*innen schon sehr häufig aktiv auf das Vorhandensein von Todeswünschen angesprochen, wohingegen es bei den Pflegenden 16,4 % waren (x^2^ = 116,55; *p* < 0,001).

Von 916 Befragten identifizierten 58 % Gesundheitspersonal als richtige Ansprechpartner*innen, um über die Zulässigkeit eines assistierten Suizides zu entscheiden. Anfragen zur Mithilfe beim FVET, assistierten Suizid oder nach Tötung auf Verlangen wurden gelegentlich oder seltener an die Teilnehmenden herangetragen. Um Mithilfe beim assistierten Suizid wurden 182/926 (19,6 %) der Teilnehmenden mehr als 10 Mal gebeten. Die Prävalenzen von Anfragen zur Mithilfe beim FVET und Tötung auf Verlangen (in Deutschland illegal) sind in Tab. 3 dargestellt. In der Subgruppenanalyse wurden 25,1 % der teilnehmenden Ärzt*innen in mehr als 10 Fällen um Mithilfe beim assistierten Suizid gebeten, unter den Pflegenden waren es 14,2 %. Für die Fragen nach Mithilfe beim FVET (Q20), assistierten Suizid (Q21) und Tötung auf Verlangen (in Deutschland illegal, Q23) ergaben sich für die verschiedenen Professionen statistisch signifikante Unterschiede (Q20 : Χ^2^ = 109.234, *p* < 0,01; Q21 : Χ^2^ = 101.697, *p* < 0,01; Q22: Χ^2^ = 85.715, *p* = 0,01) in der Häufigkeit der Konfrontation mit solchen Bitten.

**Tab. 3:** Angaben der Teilnehmenden zu Anfragen von Patient*innen um Mithilfe beim freiwilligen Verzicht auf Essen und Trinken (FVET), assistierten Suizid und Tötung auf Verlangen (in Deutschland verboten)

	Noch nie *n* [%]	Vereinzelt (≤ 2-mal) *n* [%]	Gelegentlich (3- bis 10-mal) *n* [%]	Häufig (11- bis 50-mal) *n* [%]	Sehr häufig (> 50-mal) *n* [%]
Q20: Ich bin um Mithilfe zum freiwilligen Verzicht auf Essen und Trinken gebeten worden (*n* = 925)	248 [26,8]	192 [20,8]	279 [30,2]	161 [17,4]	45 [4,9]
Q21: Ich bin schon um Mithilfe zum assistierten Suizid gebeten worden (*n* = 926)	249 [26,9]	251 [27,1]	244 [26,3]	139 [15,0]	43 [4,6]
Q23: Ich bin um Mithilfe zur Tötung auf Verlangen gebeten worden (*n* = 927)	384 [41,4]	242 [26,1]	161 [17,4]	111 [12,0]	29 [3,1]

### Suizidassistenz als professionsabhängige Aufgabe.

Inwieweit Teilnehmende die Mitwirkung an einem assistierten Suizid als mögliche Option ihrer Berufsgruppe interpretieren oder als deren immanente Aufgabe verstehen, wurde anhand zweier Fragen erfasst. 287/911 (31,6 %) befürworteten die Aussage, dass die unmittelbare Mitwirkung beim assistierten Suizid eine ärztliche Aufgabe ist. Die Aussage: „Die unmittelbare Mitwirkung beim assistierten Suizid ist eine pflegerische Aufgabe“, lehnten 689/910 (75,7 %) ab. Tab. 4 fasst die Einschätzung der Befragten nach professionsabhängiger Zuständigkeit zusammen.

**Tab. 4:** Einschätzungen der Teilnehmenden zur Professionsabhängigkeit der Suizidassistenz

	Trifft voll zu *n* [%]	Trifft eher zu *n* [%]	Weder noch *n* [%]	Trifft eher nicht zu *n* [%]	Trifft gar nicht zu *n* [%]
Q31: Die unmittelbare Mitwirkung beim assistierten Suizid *kann* eine pflegerische Aufgabe sein (*n* = 910)	77 [8,5]	287 [31,5]	101 [11,1]	259 [28,5]	186 [20,4]
Q47: Die unmittelbare Mitwirkung beim assistierten Suizid *ist* eine pflegerische Aufgabe (*n* = 910)	23 [2,5]	89 [9,8]	109 [12,0]	274 [30,1]	415 [45,6]
Q32: Die unmittelbare Mitwirkung beim assistierten Suizid *kann* eine ärztliche Aufgabe sein (*n* = 911)	186 [20,4]	423 [46,4]	103 [11,3]	134 [14,7]	65 [7,1]
Q46: Die unmittelbare Mitwirkung beim assistierten Suizid *ist* eine ärztliche Aufgabe (*n* = 911)	87 [9,6]	200 [22,0]	131 [14,4]	254 [27,9]	239 [26,2]

In der Subgruppenanalyse waren 8,8 % der teilnehmenden Ärzt*innen der Auffassung, dass die Mitwirkung beim assistierten Suizid eine pflegerische Aufgabe ist, unter den Pflegenden waren es 18,4 %. 27,7 % der Ärzt*innen und 37,6 % der Pflegenden betrachteten die unmittelbare Mitwirkung beim assistierten Suizid als ärztliche Aufgabe. Die unterschiedlichen Auffassungen waren alle signifikant (jeweils *p* < 0,01).

### Suizidprävention.

Der Aussage, Aufgabe von Palliativteams sei es, sich auf Maßnahmen zur Suizidprävention zu beschränken, stimmten 379/914 (41,5 %) der Befragten zu. 430/914 (47 %) gaben an, die Aufgabe von Palliativteams solle sich *nicht* auf Maßnahmen zur Suizidprävention beschränken. Im Hinblick auf mögliche Maßnahmen zur Suizidprävention (Mehrfachantwort möglich, *n* = 1387 Antworten gesamt) wurden vor allem eine palliativmedizinische Versorgung (*n* = 372/1387, 26,8 %), die gezielte Sedierung bei therapierefraktärem Leid (*n* = 328/1387, 23,7 %), die Beendigung lebensverlängernder Maßnahmen (*n* = 299/1387, 21,6 %) sowie eine Unterstützung beim FVET (*n* = 226/1387, 16,3 %) genannt. Weiterhin gaben 80/1387 (5,8 %) der Befragten an, dass die Weitergabe von Informationen über Suizidassistenz ein wichtiger Bestandteil suizidpräventiver Maßnahmen sei. 26/1387 (1,9 %) der Befragten (*n* = 26/1387) erachteten eine Kontaktherstellung zu Sterbehilfeorganisationen als besonders bedeutend.

Unter der Kategorie „weitere“ war es möglich, mit einem Freitext zu antworten. Es gab 48 Freitextantworten, welche in Kategorien subsummiert wurden. Hierbei wurden als Maßnahmen der Suizidprävention eine psychosozial-spirituelle Unterstützung (*n* = 20, 41,7 %), hospizliche und pflegerische Versorgung (*n* = 6, 12,5 %), Aufklärung und Beratung zu Möglichkeiten der Palliativmedizin (*n* = 11, 22,9 %) sowie ein genaues Eruieren von Todeswünschen im Rahmen intensiver Begleitung (*n* = 11, 22,9 %) genannt.

## Diskussion

Das Ziel der Umfrage war es, bei Mitgliedern der Deutschen Gesellschaft für Palliativmedizin den Wissensstand und die Haltungen zum Wunsch nach assistiertem Suizid sowie den Umgang mit Todeswünschen zu erheben. Ähnliche, z. T. monoprofessionelle Befragungen wurden im Jahr 2021 innerhalb der Deutschen Gesellschaft für Hämatologie und Medizinische Onkologie (DGHO; [10]) und 2022 in der Deutschen Gesellschaft für Psychiatrie und Psychotherapie, Psychosomatik und Nervenheilkunde (DGPPN) durchgeführt [11]. Die Rücklaufquoten der jeweiligen Online-Umfragen waren vergleichbar (DGHO: 20,8 %, DGPPN: 20,0 %, DGP: 18,0 %). Entsprechend der Verteilung innerhalb der DGP nahmen an unserer Befragung überwiegend Ärztinnen, gefolgt von weiblichen Pflegenden, an der Befragung teil. Mit einer Mehrheit von über 70 % bildete sich dabei das Meinungsbild von überwiegend erfahrenen Mitgliedern mit einer Berufserfahrung von mehr als 18 Jahren ab, wovon 57,3 % der Teilnehmenden mehr als 8 Jahre in der Palliativversorgung tätig waren. Dies ist mit anderen Befragungen vergleichbar, denn auch in denen der DGHO, bei der 80,4 % der Befragten mind. 10 Jahre in der Onkologie gearbeitet haben, sowie der DGPPN, bei der 86,0 % der Teilnehmende den Facharztstaus erreicht hatten, antworteten eher erfahrene Teilnehmende.

Trotz der aktuell intensiven öffentlichen Debatte aufgrund des o. g. Urteils des Bundesverfassungsgerichts und der Ablehnung der beiden Gesetzesentwürfe zur Regulierung der Suizidassistenz zeigten sich auch bei einem Teil der Mitglieder der DGP Wissenslücken im Hinblick auf strafrechtliche und standesrechtliche Bestimmungen. Obwohl die Mehrheit der Befragten angab, assistierten Suizid, FVET und Tötung auf Verlangen definieren zu können und das Urteil des Bundesverfassungsgerichtes aus dem Jahr 2020 zu kennen, vertrat etwa jeder 20. der Befragten fälschlich die Meinung, der assistierte Suizid wäre strafrechtlich verboten. Fast jeder 5. war fälschlich der Annahme, der assistierte Suizid sei standesrechtlich verboten. Hier besteht weiterer Aufklärungsbedarf, zumal unklar ist, ob und wann neuerliche gesetzliche Regularien im Bereich der Suizidassistenz zu erwarten sind. Für die Befragten spielen diese jedoch eine wichtige Rolle – 71,0 % der Teilnehmenden wünschen sich ein Gesetz, hauptsächlich um mehr Rechtssicherheit und eine Handlungsgrundlage zu erhalten, was letztlich dem Schutz des agierenden Gesundheitspersonals dient. In der Literatur wird dies kontrovers diskutiert, da es aufgrund der Sensibilität des Themas Suizidassistenz eine Herausforderung darstellen wird, in einer Gesetzgebung alle relevanten Aspekte und betroffenen Akteure (Patient*innen, Pflegende, Ärzt*innen etc.) angemessen zu berücksichtigen [12].

Die Hälfte der Befragten kann sich eine persönliche Mitwirkung an einem assistierten Suizid nur bei palliativ erkrankten Menschen vorstellen. 82,0 % der Befragten lehnen eine persönliche Mitwirkung unabhängig vom Gesundheitszustand ab. Der Vergleich mit Erhebungen anderer Fachgesellschaften ist aufgrund jeweils unterschiedlicher Fragestellung schwierig. So stellten 30,3 % der befragten Onkolog*innen fest, unter bestimmten Bedingungen Beihilfe zum assistierten Suizid zu leisten, vergleichbare 15,6 % konnten sich grundsätzlich eine Mitwirkung vorstellen [10]. In der Befragung der DGPPN konnten sich 44,0 % bei begrenzter Prognose und hohem Leidensdruck eine Suizidassistenz vorstellen, 8,2 % werteten bei Freiverantwortlichkeit eine Suizidassistenz immer als legitim, 19,9 % nie [11]. Vergleicht man die Zustimmungsrate zum Mitwirken bei einer Suizidassistenz mit einem Kollektiv jüngerer Ärzt*innen und gleicher Fragestellung wie in unserem Kollektiv [8], so zeigt sich mit 62,3 % im jüngeren Kollektiv eine höhere Akzeptanz in palliativen Behandlungssituationen. Mit 20,1 % konnte sogar eine deutlich höhere Zustimmung zur Suizidassistenz unabhängig vom Gesundheitszustand dokumentiert werden. Inwieweit ein Zusammenhang zwischen Berufserfahrung und Einstellung zum ärztlich assistierten Suizid besteht, wird in der Literatur kontrovers diskutiert [8, 13, 14]. Gegebenenfalls deutet sich hier eine zunehmende Haltungsänderung durch den Generationsunterschied an. In einer vorangegangenen Erhebung unter Mitgliedern der DGP aus 2017 hatten 56,0 % der teilnehmenden Ärzt*innen eine Mitwirkung an einem assistierten Suizid abgelehnt [15].

In unserer Befragung zeigte sich eine häufige bis sehr häufige Frequenz von Todeswunschäußerungen. Wesentlich ist hier der respektvolle Umgang mit der verbalisierten Not der Betroffenen, wie in der S3-Leitlinie Palliativmedizin beschrieben [5]. Dabei ist besonders ein proaktives Ansprechen von Todeswünschen relevant, was für die Betroffenen entlastend wirken kann. Trotz der häufigen Frequenz geben 25,0 % der Befragten an, ihre Patient*innen nie oder in weniger als 2 Fällen aktiv auf das Vorhandensein von Todeswünschen angesprochen zu haben. Hier zeigt sich eine bereits bekannte Unsicherheit im Umgang mit Todeswünschen auch unter Mitarbeiter*innen im Bereich der Palliativversorgung [16], welcher Weiterbildungsangebote und Schulungskonzepte [17, 18] Abhilfe leisten könnten. Zuletzt hatte die DGP 2022 in einer Stellungnahme explizit die Stärkung der Aus‑, Fort- und Weiterbildung zum Umgang mit Todeswünschen aller in der Palliativversorgung tätigen Berufsgruppen gefordert [19].

Als richtige Ansprechpartner*innen für die Zulässigkeit einer Suizidassistenz wurde in unserer Befragung Gesundheitspersonal identifiziert, ebenso wie in der Befragung eines jüngeren Ärzt*innenkollektivs [8]. Es stellt sich jedoch die Frage, in welchen Aufgabenbereich die Suizidassistenz konkret fällt. Hier ist zu unterscheiden, inwieweit diese professionsbezogen als mögliche Option oder als professionsimmanente Aufgabe interpretiert wird.

Zur ärztlichen Rolle befragt, ergibt sich ein heterogenes Bild. Dabei verstehen fast 2 Drittel der Befragten die Suizidassistenz als mögliche Aufgabe, was in der Beschaffung von Medikamenten, Wissen um Möglichkeiten der Symptomkontrolle oder der Einschätzung einer Freiverantwortlichkeit liegen könnte. Nur knapp ein Drittel betrachtete die unmittelbare Mitwirkung bei Suizidassistenz als akzeptierte ärztliche Aufgabe. Die unmittelbare Mitwirkung beim assistierten Suizid begreifen 40 % der Befragten als mögliche pflegerische Aufgabe, nur 12,0 % als akzeptierte Aufgabe. Diese Unterschiede in der Auffassung der Zuständigkeiten waren berufsgruppenbezogen in unserer Befragung statistisch signifikant. In der Befragung des rein ärztlichen Kollektivs der DGHO gaben 43,6 % an, dass der assistierte Suizid von Ärzt*innen umgesetzt werden sollte, mehr als 40 % sprachen sich für einen Einbezug von Pflegenden bei Anfragen nach assistiertem Suizid aus [10]. In der Befragung der DGPPN wurde die Suizidassistenz von 12,0 % immer und von 50,0 % unter bestimmten Umständen als ärztliche Aufgabe gesehen [11]. In beiden Befragungen wurde weder zwischen optionaler und akzeptierter Aufgabe unterschieden noch die Perspektive auf die Aufgabe der Pflegenden gelenkt. Aufgrund der unterschiedlichen Fragestellungen und damit assoziierten Interpretationsspielräume sind die Antworten jedoch nur eingeschränkt vergleichbar.

Daten zum Rollen- und Aufgabenverständnis Pflegender, speziell im Kontext des assistierten Suizids, sind bisher nur anfänglich erforscht [20]. Die vorhandene Literatur bezieht sich überwiegend auf die Auseinandersetzung mit Tötung auf Verlangen [21]. Hier sind weitere Erhebungen zum Selbstverständnis Pflegender sowie auch ein fortgesetzter (fach-)gesellschaftlicher Diskurs notwendig. Daher erfolgte bereits eine Befragung Pflegender in Nordrhein-Westfalen durch unser Studienteam.

### Suizidprävention.

Mit Verkündung des verfassungsrechtlichen Urteils im Jahr 2020, der Verabschiedung eines Antrags zur Stärkung der Suizidprävention im Jahr 2023 und der aktuell durch das Bundesministerium für Gesundheit vorgestellten Suizidpräventionsstrategie wurde der Stellenwert von präventiven Maßnahmen und Hilfsangeboten für Menschen mit Suizidwünschen unterstrichen. Hier kann eine adäquate, palliativmedizinische Versorgung der betroffenen Schwerkranken eine große Rolle spielen. Suizidwünsche waren in einer Erhebung der DGP sowie der Gruppe „Assisted Suicide Critical Incident Reporting System“ (ACSIRS) in Österreich zu ca. 30 % in körperlicher oder psychischer Symptomlast begründet [22, 23]. Unkontrolliertes Leiden oder hoher Leidensdruck waren Bedingungen oder Umstände, die zu einem möglichen Mitwirken der Befragten der DGHO bzw. zu einer Legitimation des assistierten Suizids bei Befragten der DGPPN führten. Dem tritt eine multiprofessionelle ambulante oder stationäre spezialisierte palliativmedizinische Begleitung entgegen. Mit Reduktion der Symptomlast reduzieren sich häufig Todes- und Suizidwünsche [24]. Aber auch schon eine Aufklärung über Möglichkeiten der Palliativversorgung lässt Todeswünsche in den Hintergrund treten [22]. Dies könnte erklären, warum 41,5 % der Befragten der Auffassung waren, Palliativteams sollten sich auf suizidpräventive Maßnahmen beschränken. Als häufigste suizidpräventive Maßnahme wurde in unserer Befragung die palliativmedizinische Versorgung genannt, gefolgt von einer gezielten Sedierung bei therapierefraktärem Leid am Lebensende und der Beendigung lebenserhaltender Maßnahmen. Aber auch die alleinige Weitergabe von Informationen zur Suizidassistenz wurde genannt, was die Relevanz der Aufklärung der Bevölkerung zu diesem Thema untermauert. Unbenommen der Suizidprävention durch adäquate palliativmedizinische Versorgung waren die Befragten in unserer Erhebung unentschieden, ob sich die Aufgabe von Palliativteams alleinig auf Maßnahmen zur Suizidprävention beschränken sollte. In der Befragung eines jüngeren ärztlichen Kollektivs lehnte die Mehrheit der Befragten die Beschränkung der ärztlichen Aufgabe auf Maßnahmen zur Suizidprävention ab [8]. Die zu erwartende gesetzliche Stärkung der Suizidprävention kann hier zukünftig ggf. zusätzliche Angebote zur Unterstützung unserer Patient*innen eröffnen.

### Stärken und Limitationen.

Eine Limitation dieser Studie ist die Rücklaufquote von 18,0 %, sodass die Repräsentativität der Daten bezogen auf die Gesamtheit der Fachgesellschaft eingeschränkt ist, wenngleich ähnliche Befragungen anderer Fachgesellschaften vergleichbare Rücklaufquoten aufweisen. Die Ergebnisse dieser Befragung können daher nur als Trend gewertet werden, welcher Aufschluss über mögliche Einstellungen, Haltungen und Wissen geben kann.

Außerdem ergibt sich bei einigen Fragen die Gefahr, dass die Antworten nach sozialer Erwünschtheit gegeben werden. Mehrere Fragen zielen auf die persönliche Bereitschaft zur Mitwirkung bei Suizidassistenz ab. Hierbei ergeben sich relevante Limitationen. Unter anderem wird das Antwortverhalten durch weitere Differenzierung des klinischen Szenarios (z. B. schwer versus tödlich erkrankt) in der Einschätzung einer persönlichen Mitwirkung beeinflusst, was zu einer Verzerrung der Ergebnisse führen kann [25]. Im Unterschied zu den Teilnehmenden der Pretests und der Befragung unter jungen Mediziner*innen [8] stellte die nicht näher konkretisierte Fragestellung aufgrund des individuellen Interpretationsspielraums für die Kohorte der Teilnehmenden der DGP ein Problem dar, was sich in den Freitextantworten zeigte. Die Autor*innen haben sich dennoch dazu entschlossen, die Ergebnisse dieser Fragen darzustellen. Weiterhin war es das Ziel, die Vergleichbarkeit mit der erwähnten Vorbefragung unter jungen Intensiv- und Notfallmediziner*innen zu gewährleisten, sodass möglichst wenig Änderungen der Fragestellungen vorgenommen wurden.

## Fazit

Die Untersuchung zeigt, dass bei den befragten Mitgliedern der DGP Wissenslücken in Bezug auf straf- und standesrechtliche Einordnung des assistierten Suizides bestehen. Hier ist weitere Aufklärungsarbeit notwendig. Gesundheitsfachkräfte wurden als die richtigen Ansprechpartner*innen für Todes- bzw. Suizidwünsche angegeben – weiterer Handlungsbedarf besteht hier in der Aus‑, Fort- und Weiterbildung im Umgang mit Todeswünschen. Auch in dieser Befragung bildet sich, wie in anderen Fachgesellschaften zuvor, ein Bild der eher erfahrenen Mitarbeiter*innen ab. Sie weisen im Vergleich zu jüngeren Ärzt*innen eine eher restriktive Haltung im Hinblick auf eine mögliche Suizidassistenz auf. Inwieweit hier Generationsunterschiede abgebildet werden, werden zukünftige Erhebungen vor dem Hintergrund der anhaltenden Debatte um Suizidassistenz und ihre gesetzliche Regelung in Deutschland offenbaren. In unserer Stichprobe können sich die Befragten eher eine Suizidassistenz bei palliativ erkrankten Menschen vorstellen. Als suizidpräventive Maßnahmen werden vorrangig palliativmedizinische Versorgung und gezielte Sedierung bei therapierefraktärem Leid am Lebensende angesehen.
